# Supplementary material for: An open-source framework for large-scale, flexible evaluation of biomedical text mining systems
Source: J Biomed Discov Collab. 2008 Jan 29;3:1. doi: 10.1186/1747-5333-3-1 (PMC2276192; doi:10.1186/1747-5333-3-1)
Supplement: Additional file 1 — Evaluation platform source code. The additional file contains the software evaluation framework discussed in this paper. The most current release can be downloaded from SourceForge.net [1]. See the accompanying README file for instructions on installation and use. Also included in the distribution is a collection of UIMA wrappers for some commonly used BioNLP tools and annotated corpora. [file 1747-5333-3-1-S1.zip › bionlp_uima_repository-1.0.1/docs/api/edu/uchsc/ccp/util/nlp/ei/EIFileBasedSystem.html]

EIFileBasedSystem (Center for Computational Pharmacology Util/UIMA API)


|  |  |  |
| --- | --- | --- |
| **Overview** \| **Package** \| **Class** \| **Tree** \| **Deprecated** \| **Index** \| **Help** | |  |
| PREV CLASS   **NEXT CLASS** | **FRAMES**    **NO FRAMES**     **All Classes** |  |
| SUMMARY: NESTED \| FIELD \| CONSTR \| METHOD | DETAIL: FIELD \| CONSTR \| METHOD |  |


---


## edu.uchsc.ccp.util.nlp.ei Interface EIFileBasedSystem

**All Superinterfaces:**: EISystem

**All Known Implementing Classes:**: AbGeneEISystem, KeXEISystem

---

``` public interface EIFileBasedSystem extends EISystem ```

**Author:**
:   williamb

---

| **Method Summary** | |
| --- | --- |
| `List<TextAnnotation>` | `getEntitiesFromFile(String originalTextFilename)` |
| `String` | `getOutputFilename()` |

**Methods inherited from interface edu.uchsc.ccp.util.nlp.ei.EISystem**

`getEntitiesFromString`

**Method Detail**

### getEntitiesFromFile

```
List<TextAnnotation> getEntitiesFromFile(String originalTextFilename)
```

:   **Parameters:**: `inputFilename` - - the name of the tagged file which needs to be parsed to find the gene hits **Returns:**: - ArrayList of TextAnnotations

---


### getOutputFilename

```
String getOutputFilename()
```

:   **Returns:**: - String containing the name of the file where the system's output is stored


---


|  |  |  |
| --- | --- | --- |
| **Overview** \| **Package** \| **Class** \| **Tree** \| **Deprecated** \| **Index** \| **Help** | |  |
| PREV CLASS   **NEXT CLASS** | **FRAMES**    **NO FRAMES**     **All Classes** |  |
| SUMMARY: NESTED \| FIELD \| CONSTR \| METHOD | DETAIL: FIELD \| CONSTR \| METHOD |  |


---
